# Investigation of the transforming growth factor-beta 1 signalling pathway as a possible link between hyperphosphataemia and renal fibrosis in feline chronic kidney disease

**DOI:** 10.1016/j.tvjl.2020.105582

**Published:** 2021-01

**Authors:** J.S. Lawson, H.M. Syme, C.P.D. Wheeler-Jones, J. Elliott

**Affiliations:** a Comparative Biomedical Sciences, The Royal Veterinary College, Royal College Street, London, UK; b Clinical Sciences and Services, The Royal Veterinary College, Hawkshead Lane, North Mymms, Hatfield, Herts, UK

**Keywords:** Cat, CKD, Phosphate, Renal

## Abstract

Chronic kidney disease (CKD) is common in geriatric cats, and is characterised in the majority of cases by tubulointerstitial inflammation and fibrosis. Hyperphosphataemia is a frequent complication of CKD and is independently associated with severity of renal fibrosis and disease progression. Transforming growth factor-beta 1 (TGF-β1) signalling is thought to be a convergent pathway which mediates the progression of renal fibrosis in CKD. The aims of this study were to explore the interaction between increased extracellular phosphate and the TGF-β1 signalling pathway by investigating: (a) the effect of a commercially available, phosphate-restricted, diet on urinary TGF-β1 excretion in cats with CKD; and (b) the role of increased extracellular phosphate in regulating proliferation, apoptosis, and expression of genes related to TGF-β1 signalling and extracellular matrix (ECM) production in feline proximal tubular epithelial cells (FPTEC) and cortical fibroblasts from cats with azotaemic CKD (CKD-FCF).

The dietary intervention study revealed no effect of dietary phosphate restriction on urinary active TGF-β1 excretion after 4–8 weeks (*P* = 0.98), despite significantly decreasing serum phosphate (*P* < 0.001). There was no effect of increased growth media phosphate concentration (from 0.95 mM to 2 mM and 3.5 mM) on proliferation (*P* = 0.99) and apoptotic activity in FPTEC (*P* = 0.22), or expression of genes related to ECM production and the TGF-β1 signalling pathway in FPTEC and CKD-FCF (*P* > 0.05). These findings suggest the beneficial effects of dietary phosphate restriction on progression of feline CKD may not occur through modulation of renal TGF-β1 production, and do not support a direct pro-fibrotic effect of increased extracellular phosphate on feline renal cells.

## Highlights

• Chronic kidney disease (CKD) is associated with development of hyperphosphataemia.
• Severity of renal fibrosis has been correlated with degree of hyperphosphataemia.
• Transforming growth factor-β1 (TGF-β1) is a major pro-fibrotic mediator in CKD.
• A phosphate restricted diet did not affect urinary active TGF-β1 excretion in cats.
• Increased extracellular phosphate had no pro-fibrotic effect on feline renal cells.

## Introduction

Chronic kidney disease (CKD) is common in geriatric cats, and is pathologically characterised in the majority of cases by chronic tubulointerstitial inflammation and fibrosis (Lulich et al., 1992, Chakrabarti et al., 2013). The mechanisms behind this progressive fibrotic change, which features excessive accumulation of extracellular matrix (ECM) proteins within the interstitium, are not completely understood (Brown et al., 2016). The activation of renal fibroblasts towards the myofibroblast phenotype, which can be recognised by gain of alpha-smooth muscle actin (α-SMA) microfilament expression, is likely a key event (Sawashima et al., 2000). It is thought that renal tubular epithelial cell damage and death in CKD leads to continued production of pro-inflammatory/pro-fibrotic cytokines, and perpetuation of the wound-healing response by myofibroblasts rather than its resolution (Moll et al., 2013). The best-characterised pro-fibrotic mediator in both feline and human CKD is the cytokine transforming growth factor-beta 1 (TGF-β1), alongside its downstream effector connective tissue growth factor (CTGF; Meng et al., 2016). Urinary active TGF-β1 excretion has been correlated with histopathological grade of inflammation and fibrosis in cats with CKD, and increases over time in cats with progressive disease (Lawson et al., 2016). In vitro, TGF-β1 induces apoptosis and a pro-fibrotic phenotype consistent with partial epithelial-to-mesenchymal transition (EMT) in cultured feline tubular epithelial cells (FPTEC), recognised by downregulation of E-cadherin mRNA expression and upregulation of N-cadherin, TGF-β1, CTGF, collagen type I alpha-1 (Col1α1) and fibronectin mRNA expression (Lawson et al., 2018a). In addition, cultured feline cortical fibroblasts exposed to TGF-β1 transition towards the myofibroblast phenotype, upregulating expression of α-SMA mRNA and genes related to ECM production such as Col1α1 and fibronectin (Lawson et al., 2018b). Factors which upregulate TGF-β1 signalling are therefore likely to contribute towards progression of renal fibrogenesis.

Hyperphosphataemia is a common complication of CKD in both cats and humans, and is a known risk factor for the progression of CKD and mortality (Chakrabarti et al., 2012, Kuro-o, 2013). Although hyperphosphataemia may be a consequence of progressive loss of renal function, phosphate is associated with progression of kidney disease independent of glomerular filtration rate in humans (Zoccali et al., 2011), while cats with experimentally-induced CKD fed high phosphate diets develop more severe renal lesions (Ross et al., 1982), suggesting that increased serum phosphate might be causally implicated in the risk of renal disease progression. Historically, the deleterious effects of increased serum phosphate on renal function were most commonly attributed to phosphate-induced calcification of the renal parenchyma. However, in one large histopathological study the severity of the hyperphosphataemia was correlated with the severity of interstitial fibrosis in cats, but not with the presence of tubular mineralisation (Chakrabarti et al., 2013). This suggests an association between hyperphosphataemia and tubulointerstitial fibrosis in cats which may not involve parenchymal mineralisation, but which occurs via some other mechanism. There is evidence from previous studies that upregulation of TGF-β1 signalling could represent a possible link. Hyperphosphataemia is correlated with increased renal transglutaminase-2 (TG-2) expression in cats, which is an activator of the TGF-β1 signalling pathway (Sanchez-Lara et al., 2015). Previous in vitro studies have also found that increased extracellular phosphate may promote apoptosis and upregulation of TGF-β1 signalling in mouse tubular cells, as well as increasing production of ECM components and expression of α-SMA in human renal fibroblasts (Sakaguchi et al., 2015, Tan et al., 2016). Although the associations of increased extracellular phosphate with progressive loss of function and more severe pathology in the feline kidney are well documented, there are currently no published studies examining the effects of increased extracellular phosphate on feline renal cells in vitro where mechanisms could be explored.

We hypothesised that increased extracellular phosphate may modulate interstitial fibrosis in cats with CKD via upregulation of the TGF-β1 signalling pathway. We aimed to assess the effect of hyperphosphataemia on TGF-β1 signalling in vivo by investigating the effect of a commercially available, phosphate-restricted diet on urinary active TGF-β1 excretion in cats with CKD. Urinary active TGF-β1 was measured as this form of the cytokine has a short plasma half-life, and thus urinary concentrations are considered likely to originate locally (Wakefield et al., 1990). Furthermore, we aimed to investigate the role of increased extracellular phosphate in regulating proliferation, apoptosis, and expression of genes related to TGF-β1 signalling and ECM production in isolated FPTEC from cats without azotaemia and feline cortical fibroblasts from cats with azotaemic CKD (CKD-FCF).

## Materials and methods

### Dietary intervention study

Cases were obtained by searching the electronic patient record of two first-opinion geriatric cat clinics for cats diagnosed with azotaemic CKD between January 2010 and December 2015. Renal azotaemia was defined as a plasma creatinine concentration >177 μmol/L (laboratory reference interval: 20.0–177.0 μmol/L) with concurrent urine specific gravity <1.035. Newly diagnosed cats with urine samples both prior to and 4–8 weeks after commencing a commercially available, phosphate-restricted, renal diet (Feline Renal Support, Royal Canin Veterinary Diet, Royal Canin) were identified, and this group was further subdivided into cats hyper- or normophosphataemic for their International Renal Interest Society (IRIS) stage at baseline (Elliott, 2007). Cats could be fed a mixture of the wet and dry formulation of this diet. The phosphorous concentration of the renal diet for the duration of the study was 0.77 g/Mcal in the dry formulation and 0.9 g/Mcal in the wet formulation. A comparator group was formed from cats that did not accept the renal diet, and that were fed a variety of non-phosphate-restricted, commercially available, diets. The 4–8 week study period was chosen as a previous study demonstrated that dietary phosphate restriction significantly decreases the plasma concentration of phosphate, as well as phosphaturic hormones fibroblast growth factor-23 (FGF-23) and parathyroid hormone (PTH), over this timeframe (Geddes et al., 2013). Groups were matched so as no measured clinicopathological variable, aside from plasma phosphate, differed at baseline (Table 1). Cats with CKD that progressed during the study period (as indicated by a >25% increase in plasma creatinine concentration) were excluded, as were cats with hyperthyroidism (plasma total thyroxine >40 nmol/L), urinary tract infection or other systemic disease (except hypertension).

**Table 1:** Summary statistics for cats in the dietary intervention study. Values reported represent the median (range).

	Hyperphosphataemic group (*n* = 14)		Normophosphataemic group (*n* = 12)		Comparator group (*n* = 15)	
	Baseline	4–8 weeks	Baseline	4–8 weeks	Baseline	4–8 weeks
Age (years)	15.0 (9.0–20.1)	15.1 (9.1–20.2)	14.0 (9.0–19.1)	14.1 (9.1–19.2)	15.1 (10.0–19.0)	15.2 (10.1–19.1)
Creatinine (μmol/L)	224.0 (176.2–462.3)	227.5 (155.1–359.2)	240.6 (175.8–317.8)	217.2 (174.0–359.0)	215.6 (177.3–269.3)	208.4 (166.2–289.3)
Urea (mmol/L)	17.7 (8.9–29.4)	21.1 (10.0–29.5)	18.4 (12.6–32.5)	15.5 (9.3–23.9)	16.3 (8.8–25.3)	17.5 (9.9–30.2)
Phosphorous (mmol/L)	1.77 (1.46–2.37)	1.19 (0.83–1.80)	1.22 (0.78–1.57)	1.19 (0.64–1.54)	1.30 (0.93–2.10)	1.28 (0.90–1.89)
Total calcium (mmol/L)	2.48 (2.28–2.67)	2.56 (2.37–2.84)	2.49 (2.28–2.92)	2.53 (2.31–3.29)	2.56 (2.22–2.75)	2.59 (2.30–2.81)
Total protein (g/L)	76.9 (70.1–92.9)	78.4 (70.8–92.6)	80.6 (63.7–92.2)	77.6 (62.0–91.7)	77.1 (69.9–83.8)	76.5 (71.6–84.8)
Albumin (g/L)	31.1 (26.3–36.4)	30.9 (28.1–35.1)	31.1 (22.1–37.3)	31.1 (22.5–36.9)	31.8 (27.9–37.5)	32.1 (26.8–39.2)
Globulin (g/L)	46.7 (40.5–59.4)	45.6 (39.7–59.4)	52.0 (32.0–59.9)	48.4 (29.7–60.0)	45.9 (34.4–73.7)	45.0 (35.5–57.1)
PCV (%)	34 (21–43)	31 (22–40)	33 (23–44)	33 (24–40)	36 (30–46)	35 (26–45)
UPC	0.19 (0.05–2.46)	0.24 (0.03–2.62)	0.17 (0.03–0.37)	0.14 (0.03–0.27)	0.13 (0.06–0.36)	0.13 (0.07–0.50)
USG	1.016 (1.010–1.020)	1.017 (1.010–1.030)	1.020 (1.020–1.030)	1.019 (1.020–1.030)	1.018 (1.010–1.030)	1.018 (1.010–1.030)
Weight (kg)	3.8 (2.5–4.5)	3.9 (2.6–4.7)	3.8 (2.4–5.1)	4.0 (2.8–5.1)	4.0 (2.8–5.1)	4.1 (2.8–5.2)
aTGF-β1:UCr (pg/mg)	33.5 (1.5–154.9)	35.99 (0.9–160.4)	19.5 (10.2–25.9)	19.1 (1.3–39.9)	16.3 (2.8–48.8)	28.0 (3.4–57.3)

PCV, packed cell volume; UPC, urine protein creatinine ratio; USG, urine specific gravity; aTGF-β1:UCr, active transforming growth factor-beta 1 to urinary creatinine ratio.

Blood and urine samples were obtained by jugular venepuncture and cystocentesis respectively, and stored at 4 °C for 1–4 h prior to sample processing. Urine sediment examination was performed in-house. Plasma biochemistry, urine creatinine concentration and urine protein concentration were measured at an external laboratory (IDEXX Laboratories) using a AU5800 clinical chemistry analyser (Beckmann Coulter). Residual samples of urine and plasma were retained for research use with owner informed consent. The project protocol, owner information sheet and consent forms were approved by the Royal Veterinary College's Ethics and Welfare Committee (Approval number, URN 2013 1258; Approval date 2 December, 2013). All urine samples were centrifuged at 2016 × *g*, at 4 °C for 10 min Subsequently the supernatant was removed and stored at −20 °C for 1–14 days before long term storage at −80 °C.

A sandwich ELISA (LEGEND MAX Free Active TGF-β1 ELISA Kit, BioLegend) previously validated for use in cats was used for the quantification of active TGF-β1 (aTGF-β1) in feline urine (Lawson et al., 2016). The assay was performed according to the manufacturer's protocol. TGF-β1 concentrations were normalised as a urinary aTGF-β1 to urine creatinine ratio (aTGF-β1:UCr).

### Isolation and culture of FPTEC and CKD-FCF

Feline renal tissue was obtained with owner informed consent during post-mortem examinations, and FPTEC and CKD-FCF were isolated as previously described (Lawson et al., 2018a, Lawson et al., 2018b). FPTEC were derived from the kidneys of cats without azotaemia, and CKD-FCF were derived from the kidneys of cats with azotaemic CKD. Experiments utilising FPTEC took place in serum-free medium (pH 7.4) supplemented with 10 ng/mL epidermal growth factor (Invitrogen), 36 ng/mL dexamethasone (Sigma-Aldrich), 2 ng/mL triiodothyronine (Sigma-Aldrich), 1% insulin-transferrin-selenium (Thermo Fisher Scientific) and 1% antimicrobial solution (Gibco Antibiotic-Antimycotic (100x); Thermo Fisher Scientific). Experiments utilising CKD-FCF were performed in reduced serum DMEM-F12 (pH 7.4) containing 3% foetal bovine serum (FBS; Thermo Fisher Scientific). All experiments were performed using cells at passages 1 or 2. Experiments were performed in quadruplicate, where each experimental repeat represented a separate isolation from an individual cat. Phosphate was supplemented, from a standard concentration of 0.95 mM, by the addition of a pH 7.4 stock solution of sodium phosphate monobasic/sodium phosphate dibasic (NaH_2_PO_4_/Na_2_HPO_4_; Sigma–Aldrich).

### Effect of increased extracellular phosphate on FPTEC proliferation

FPTEC proliferation was assessed by counting cells after incubation for 72 h in medium supplemented to a total concentration of 3.5 mM inorganic phosphate. Addition of TGF-β1 (10 ng/mL; BioLegend) to growth media containing a standard concentration of phosphate was used as a positive control for apoptosis. Cells were seeded at 5000 cells/well into 96-well cell culture plates (Nunc), with six technical replicates for each treatment. After 72 h cells were washed with Dulbecco's phosphate buffered saline (DPBS; Sigma–Aldrich) before fixation (4% formaldehyde, 15 min). Cells were then washed with DPBS before incubation with 1 μg/ml of 4′,6-diamidino-2-phenylindole (DAPI; Sigma-Aldrich) diluted in DPBS for 10 min in the dark, followed by a final wash in DPBS. Low power images (2.5×) of the wells were collected using the DM4000B upright microscope (Leica). The number of cells per image was counted using freely available software (ImageJ). Data are presented as fold change in cell number in comparison to cells incubated in unmodifed growth medium.

### Effect of increased extracellular phosphate on apoptotic activity in FPTEC

The effect of increased (3.5 mM) extracellular phosphate concentration on apoptotic activity in FPTEC after incubation for 72 h was determined using a commercial luminescent assay for caspase 3/7 activity (Caspase-Glo® 3/7 Assay, Promega) as per manufacturer's instructions. Luminescence was measured using a microplate reader (Infinite M200, Tecan). TGF-β1 (10 ng/mL) was used as a positive control for apoptosis. This assay was performed on cells seeded into white-walled 96-well plates (Nunc) and cultured to confluence, with six technical replicates for each treatment. Data are presented as fold change in comparison to cells incubated in unmodifed growth medium.

### Effect of increased extracellular phosphate on pro-fibrotic gene expression in FPTEC and CKD-FCF

Confluent monolayers of FPTEC were incubated in the presence of 2 mM and 3.5 mM inorganic phosphate for 24, 72, and 168 h (3.5 mM only), with growth medium containing 0.95 mM inorganic phosphate as a control. These concentrations of phosphate were chosen as representative of plasma phosphate concentrations in cats with moderate (2 mM) and severe (3.5 mM) renal dysfunction (Barber and Elliott, 1998). Due to limited availability, experiments on CKD-FCF were only performed at a single incubation duriation (72 h) and phosphate concentration (3.5 mM). This experiment was also performed using confluent monolayers of cells, and growth medium containing 0.95 mM inorganic phosphate was used as a control. Cells were lysed and RNA was extracted from cells using a column based kit (GenElute Mammalian Total RNA Miniprep Kit, Sigma–Aldrich). Messenger RNA (mRNA) templates were reverse transcribed to complementary DNA (cDNA) using a commercially available kit (Omniscript RT, Qiagen), oligo dT primer (MWG Eurofins) and 1 U/mL ribonuclease inhibitor (RNaseOUT, Life Technologies). Gene expression was quantified by quantitative PCR in 96-well plates (FrameStar, Fortitude) using a commercially available SYBR green-based assay (SYBR® Green JumpStar Taq ReadyMix™, Sigma–Aldrich), and was performed in a CFX Connect™ Real-Time PCR Detection System (Bio-Rad).

Collagen type I α1, CTGF, TG-2, fibronectin, E-cadherin, N-cadherin, α-SMA and TGF-β1 gene expression were analysed by reverse transcriptase-quantitative PCR (RT-qPCR) and normalised to a combination of two reference genes (*fGAPDH* and *fRPS7*) using previously published primer sequences (Lawson et al., 2018a). Genes of interest were selected due to prior studies demonstrating their involvement in the TGF-β1 signalling pathway and/or renal fibrosis in cats (Sanchez-Lara et al., 2015, Lawson et al., 2016, Lawson et al., 2018a, Lawson et al., 2018b). Data are expressed as mean fold change relative to the unmodified controls.

### Statistical analysis

Statistical analysis was carried out using commercial software (IBM SPSS Statistics for Windows, Version 20.0; GraphPad Prism software version 6.0). Urinary aTGF-β1 demonstrated a non-Gaussian distribution and was log-transformed for analysis. Difference in urinary aTGF-β1:UCr between hyperphosphataemic and normophosphataemic cats at baseline was assessed using the Student's *t*-test with Welch's correction. Effect of dietary phosphate restriction on plasma phosphorous was assessed using the two-way repeated measures analysis of variance (ANOVA). Effect of dietary phosphate restiction on log-transformed aTGF-β1:UCr was assessed using a mixed effects model. Net change in aTGF-β1:UCr from pre- to post-dietary phosphate restriction was calculated for individual cats, and net change compared between groups using the Brown–Forsythe ANOVA test. Equality of variance between groups was assessed using the *F*-test or Brown–Forsythe test depending on number of groups. Significance was set at *P* < 0.05. Results are reported as median values (range). Statistical significance in the cell viability, proliferation and gene expression experiments was evaluated by Student's *t*-test or one-way ANOVA with post hoc Dunnett's test depending on number of groups.

## Results

### Dietary intervention study

Forty-one cats were included in the dietary intervention study: 26 in the dietary phosphate restriction group (*n* = 14 hyperphosphataemic; *n* = 12 normophosphataemic) and 15 in the comparator group. Summary statistics for these groups can be found in Table 1. The dietary phosphate restriction significantly decreased median plasma phosphate after 4–8 weeks in the hyperphosphataemic group (baseline, 1.8 mmol/L; 4–8 weeks, 1.2 mmol/L; *P* < 0.001) but not the normophosphataemic group (baseline, 1.2 mmol/L; 4–8 weeks, 1.2 mmol/L; *P* = 0.89). There was no change in plasma phosphate in the comparator group (baseline, 1.3 mmol/L; 4–8 weeks, 1.3 mmol/L; *P* = 0.81).

**Fig. 1:**
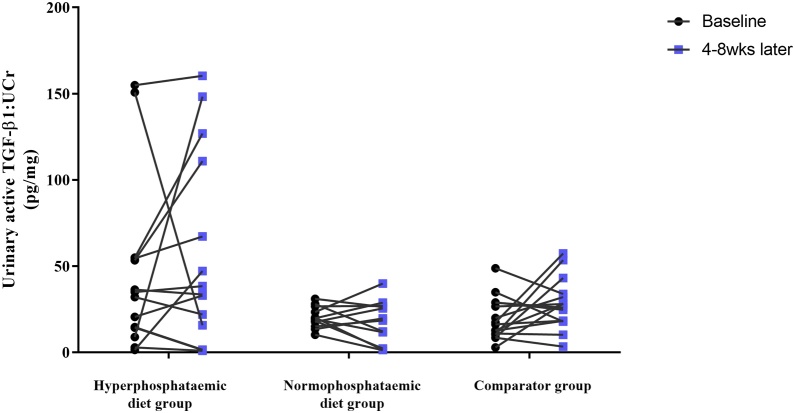
Scatterplot illustrating urinary active transforming growth factor-beta 1: urinary creatinine ratio (aTGF-β1:UCr) of cats from the renal diet study. The groups are formed of cats newly diagnosed with chronic kidney disease which were: (1) hyperphosphataemic at baseline and fed renal diet for 4–8 weeks (hyperphosphataemic plus diet group; *n* = 14); (2) normophosphataemic at baseline and fed renal diet for 4–8 weeks (normorphosphataemic plus diet group; *n* = 12); and (3) not changed onto renal diet after diagnosis irrespective of baseline serum phosphate (comparator group; *n* = 15). Black circles represent the baseline aTGF-β1:UCr and blue squares represent the aTGF-β1:UCr after 4–8 weeks. Mixed effects model analysis found no effect of phosphate-restricted renal diet on aTGF-β1:UCr (*P* = 0.38).

There was no difference between aTGF-β1:UCr in hyperphosphataemic and normophosphataemic animals at baseline (*P* = 0.17), although there was significantly more variability within the hyperphosphataemic group (*P* = 0.002). There was no effect of dietary phosphate restriction on aTGF-β1:UCr when comparing cats in the dietary phosphate restriction group (baseline, 20.4 pg/mg [1.5–154.9 pg/mg]; 4–8 weeks, 25.9 pg/mg [0.9–160.4 pg/mg]) and comparator group (baseline, 16.3 pg/mg [2.8–48.8 pg/mg]; 4–8 weeks, 28.0 pg/mg [3.4–57.3 pg/mg]; *P* = 0.98). Median net change in aTGF-β1:UCr was close to zero, and did not differ between groups (dietary phosphate restriction group, 1.8 pg/mg [−135.1 to 139.3 pg/mg]; comparator group, 2.1 pg/mg [−17.0 to 43.2 pg/mg]; *P* = 0.73). There remained no difference when cats in the dietary phosphate restriction group were subdivided into hyperphosphataemic (baseline, 33.5 pg/mg [1.5–154.9 pg/mg]; 4–8 weeks, 36.0 pg/mg [0.9–160.4 pg/mg]) and normophosphataemic (baseline, 19.5 pg/mg [10.2–25.9 pg/mg]; 4–8 weeks, 19.1 pg/mg [1.3–39.9 pg/mg]; *P* = 0.38; Fig. 1). Median net change in aTGF-β1:UCr did not differ between the hyperphosphataemic (4.5 pg/mg [−135.1 to 139.3 pg/mg]) and normophosphataemic groups (−2.3 pg/ml [−17 to 43.2 pg/mg]; *P* = 0.58). There was significantly more variability in the net change in aTGF-β1:UCr within the hyperphosphataemic group (*P* = 0.002).

### Cell proliferation and apoptosis in FPTEC

**Fig. 2:**
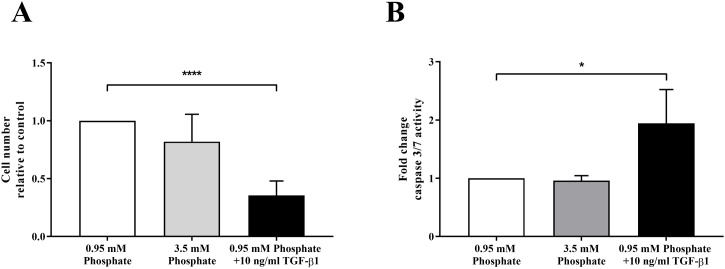
The effect of increasing growth media inorganic phosphate from a ‘normophosphataemic’ concentration of 0.95 mM to a ‘hyperphosphataemic’ concentration of 3.5 mM on feline proximal tubular epithelial cell proliferation (A) and apoptotic activity, as measured by caspase 3/7 activity (B). Addition of transforming growth factor-beta 1 (TGF-β1; 10 ng/ml) to the ‘normophosphataemic’ growth media was used as a positive control for apoptosis. This revealed no effect of 3.5 mM phosphate in the growth media on either proliferation (*P* = 0.99) or apoptotic activity (*P* = 0.22). Data were analysed using the one-way ANOVA. Columns represent the mean and error bars show the standard deviation (*n* = 4 for the proliferation experiment, and *n* = 3 for the caspase 3/7 activity measurements). *P < 0.05 and ****P < 0.0001.

There was no effect of increasing extracellular phosphate from 0.95 mM (‘normophosphataemic’ conditions) to 3.5 mM (‘severe hyperphosphataemic’ conditions) on FPTEC proliferation (*P* = 0.22) or apoptotic activity (*P* = 0.99) after 72 h incubation (Fig. 2)

### Increased extracellular phosphate and pro-fibrotic gene expression in FPTEC and CKD-FCF

**Fig. 3:**
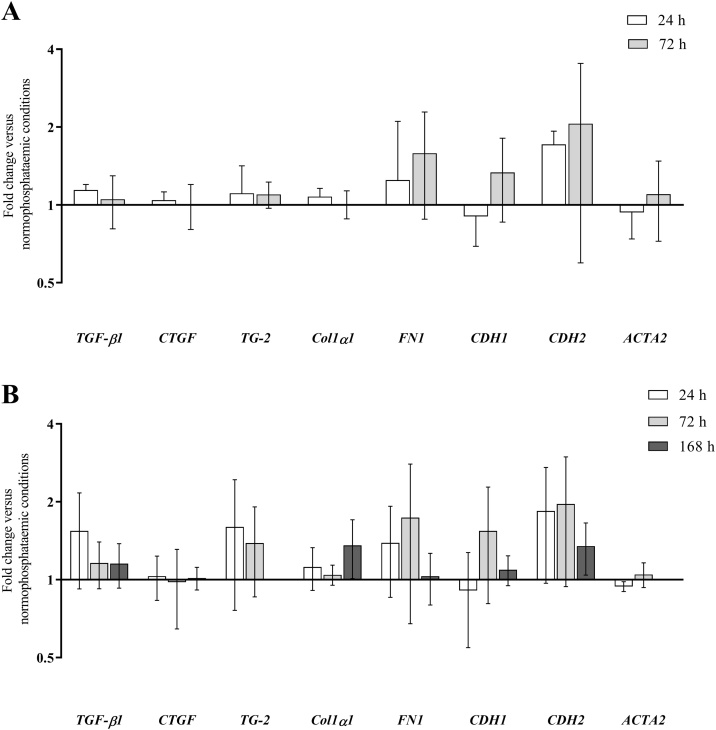
The effect of increasing growth media inorganic phosphate from a ‘normophosphataemic’ concentration of 0.95–2 mM (A) and 3.5 mM (B) on the expression of genes associated with transforming growth factor-beta 1 (TGF-β1) signalling, the extracellular matrix and epithelial-to-mesenchymal transition in feline proximal tubular epithelial cells was assessed by reverse transcriptase-quantitative PCR. There was no effect of increasing growth media phosphate concentrations to 2 mM for 24 and 72 h, or to 3.5 mM for 24, 72 and 168 h on expression of any of the genes assessed. Data were analysed using the one-way ANOVA. Columns represent the mean and error bars show the standard deviation (*n* = 4). *TGF-β1*, TGF-β1 gene; *CTGF*, connective tissue growth factor gene; *TG-2*, tissue transglutaminase-2 gene; *Col1α1*, collagen type I alpha-1 gene; *FN1*, fibronectin gene; *CDH1*, E-cadherin gene; *CDH2*, N-cadherin gene; *ACTA2*, alpha-smooth muscle actin gene.

**Fig. 4:**
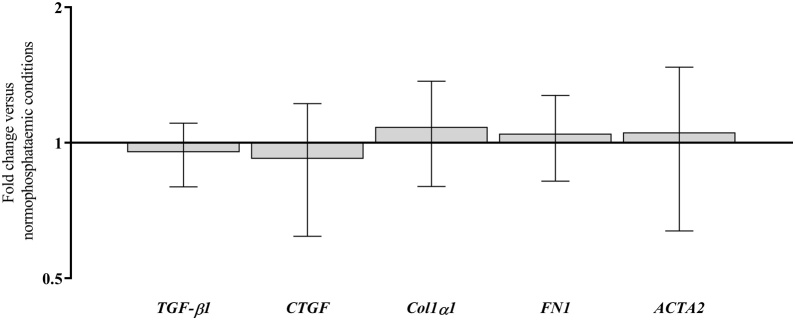
The effect of increasing growth media inorganic phosphate from a ‘normophosphataemic’ concentration of 0.95 mM to 3.5 mM phosphate on expression of genes associated with transforming growth factor-beta 1 (TGF-β1) signalling and myofibroblast activation was assessed in feline cortical fibroblasts from cats with chronic kidney disease after 72 h. This revealed no effect of increased extracellular phosphate on target gene expression. Data were analysed using the one-way ANOVA. Columns represent the mean and error bars show the standard deviation (*n* = 4). *TGF-β1*, TGF-β1 gene; *CTGF*, connective tissue growth factor gene; *Col1α1*, collagen type I alpha-1gene; *FN1*, fibronectin gene; *ACTA2*, alpha-smooth muscle actin gene.

Phosphate at 2 mM or 3.5 mM had no significant effect on expression of Col1α1, CTGF, TG-2, fibronectin, E-cadherin, N-cadherin, α-SMA or TGF-β1 mRNA in FPTEC over the course of 24, 72, or 168 h incubation periods (Fig. 3). *P*-values for all comparisons can be found in Appendix A: Supplementary material Table S1. There was intra-experimental variability in the expression of some of these genes in response to increased phosphate concentrations, particularly N-cadherin and fibronectin, which was more apparent at 3.5 mM. There was no effect of 3.5 mM phosphate on the expression of Col1α1, fibronectin, α-SMA, CTGF, or TGF-β1 mRNA in the CKD-FCF after 72 h incubation (Fig. 4). *P*-values for can be found in Appendix A: Supplementary material Table S2.

## Discussion

Hyperphosphataemia and high phosphate diets have previously been associated with progression of kidney disease and severity of interstitial fibrosis in naturally-occurring and experimentally-induced feline CKD (Ross et al., 1982, Chakrabarti et al., 2012, Chakrabarti et al., 2013). TGF-β1 is a well characterised pro-fibrotic mediator, and urinary aTGF-β1 excretion is associated with histopathological grade of fibrosis in cats with CKD (Lawson et al., 2016). This study tested the hypothesis that hyperphosphataemia may mediate its deleterious effects on the renal parenchyma through upregulation of the TGF-β1 signalling pathway.

Dietary phosphate restriction is a mainstay of therapy for the treatment of CKD in cats and the only therapeutic intervention where there is evidence for a beneficial effect on all-cause mortality (Elliott et al., 2000, Ross et al., 2006). In the present study, feeding phosphate-restricted renal diet to cats with CKD significantly decreased plasma phosphate in cats that were hyperphosphataemic at baseline, but failed to have any effect on urinary aTGF-β1 excretion, and the median net change in aTGF-β1 in all groups after 4–8 weeks was small. Despite this lack of association, there was considerably more variability in urinary aTGF-β1 excretion within the hyperphosphataemic group, which could suggest an alteration of TGF-β1 signalling in this population. Although there are no comparable clinical studies from human medicine, there is evidence of a link between hyperphosphataemia and TGF-β1 signalling from experimental rodent models, where high phosphate diets upregulate renal TGF-β1 mRNA expression, and low phosphate/protein diets reduce renal TGF-β1 expression (Okuda et al., 1991, Shen et al., 2016). As the current study was performed using clinical cases, urinary aTGF-β1 was used as a surrogate for renal TGF-β1 expression. This was chosen as the active form of TGF-β1 has a short plasma half-life and is therefore likely to originate locally from the urinary tract, and urinary aTGF-β1 has previously been correlated with evidence of fibrosis and inflammation in feline renal tissue (Wakefield et al., 1990, Lawson et al., 2016). In addition, measurement in urine is considered more appropriate than in serum or plasma due to possible contamination with platelet-derived TGF-β1 in the latter case (Coupes et al., 2001). However, it is possible that urinary aTGF-β1 did not truly reflect renal parenchymal cytokine concentrations, and renal biopsy would be required to definitively investigate expression. It is possible that the chosen period of 4–8 weeks used in the present study was of insufficient length to observe an effect of diet on aTGF-β1 excretion. However, this period was chosen as feeding of a phosphate-restricted diet has been demonstrated to decrease plasma phosphate, PTH and FGF-23 over this timeframe (Geddes et al., 2013). In addition, studies in humans with CKD have noted an effect of interventions such as dietary alteration, cholecalciferol and angiotensin receptor blockers on urinary TGF-β1 after similar periods (Agarwal et al., 2002, Kim et al., 2011, Goraya et al., 2012). Finally, it is possible that in cats the beneficial effects of a phosphate-restricted renal diet on the progression of CKD, and renal fibrosis in particular, are not mediated through the TGF-β1 pathway, or that the effects are too small to be detected by the size of the current study. Larger studies would be required in future to more definitively rule out an association.

The present study modelled the effect of hyperphosphataemia on the feline kidney by exposing FPTEC and CKD-FCF to concentrations of phosphate equivalent to plasma concentrations seen in cats with moderate to severe renal dysfunction. In this study, the hyperphosphataemic cats had a median serum phosphate of 1.77 mmol/L at baseline, roughly approximating the cell culture wells containing 2 mM of extracellular phosphate, which fell to 1.19 mmol/L after 4–8 weeks on diet, a concentration closer to the 0.95 mM found in the control wells. Tubular cell proliferation and apoptosis were assessed as alterations in these processes are considered integral to the maladaptive repair seen in CKD. No modulatory effect of increased extracellular phosphate on FPTEC proliferation was identified. To the author's knowledge, there are no comparable studies examining the effect of increased inorganic phosphate alone on tubular epithelial cell proliferation in other species, but one previous study reported no effect of calcium phosphate crystals on proliferation of a porcine proximal tubular cell line (LLC-PK1) and canine collecting duct cell line (MDCK; Aihara et al., 2003). This suggests hyperphosphataemia does not impair tubular repair after injury, which is accomplished by tubular epithelial cell proliferation. Future studies could be performed to assess the proliferative and apoptotic response of CKD-FCF to increased extracellular phosphate; however, there were insufficient cells isolated to perform these experiments as part of the present study.

Apoptotic tubular cells are implicated in the progression of renal fibrosis due to their release of pro-fibrotic and pro-inflammatory mediators (Mao et al., 2008) The lack of effect of increased extracellular phosphate concentrations on apoptotic activity (as assessed by caspase 3/7 activity measurements) was unexpected. In previous studies, increased extracellular phosphate has been associated with activation of the mitochondrial apoptotic pathway (Sakaguchi et al., 2015). Additionally, experimental mouse models of extreme hyperphosphataemia exhibit a markedly increased number of apoptotic cells within the kidney in comparison to normophosphataemic controls (Ohnishi and Razzaque, 2010). The lack of effect of increased phosphate on cell proliferation and apoptosis in this study suggest that feline tubular cells may be less sensitive to the pro-apoptotic effect of increased extracellular phosphate, at least in vitro, than those derived from mice.

The current study also revealed that there was no effect of increased concentrations of extracellular phosphate on expression of target genes associated with TGF-β1 signalling (TGF-β1, CTGF, TG-2), ECM production (Col1α1, fibronectin), EMT (N-cadherin, E-cadherin) or myofibroblast induction (α-SMA). The results from the CKD-FCF experiment are contrary to previous studies utilising rat and human renal fibroblast cell lines, where extracellular phosphate concentrations in the range of 2.5–5 mM have been reported to increase collagen, fibronectin and α-SMA gene expression (Chen et al., 2012, Tan et al., 2016). The previously published evidence for phosphate-mediated pro-fibrotic alterations in renal tubular epithelial cells is less clear. Extracellular phosphate concentrations of 2 mM have previously been reported to enhance TGF-β1 mRNA expression in mouse tubular cells; however, in another study utilising rat tubular cells, no effect of increased extracellular phosphate on transcription of genes related to TGF-β1 signalling was reported (Sun et al., 2014, Sakaguchi et al., 2015). Thus, there may be significant variation in the response to increased extracellular phosphate depending on the tubular cell type, species of origin, and culture conditions. One hypothesis for the lack of effect of increased extracellular phosphate on the FPTEC in this study may be the lack of serum present in the experimental medium. In high phosphate medium containing serum, phosphate forms insoluble nanoparticles known as calciprotein particles, which have been postulated to mediate the effects of increased extracellular phosphate concentrations in vitro (Kuro-o, 2013). These particles would not have been able to form in FPTEC experiments; however, the CKD-FCF experimental culture medium did contain 3% foetal bovine serum and still no effect was observed under these conditions.

The findings of the present study suggest that hyperphosphataemia may not directly induce pro-fibrotic alterations in feline renal cells. It is therefore possible that the observed link between hyperphosphataemia and renal interstitial fibrosis may be attributable to some other mechanism, such as resultant increases in the phosphaturic hormones FGF-23 and PTH. FGF-23 has been found to supplant phosphate in multivariable analyses of predictors of survival time in cats with CKD (Geddes et al., 2015), and has been reported to upregulate of components of the TGF-β1 signalling pathway in mouse models (Dai et al., 2012). Additionally, one study of human patients found that PTH was an independent predictor of interstitial fibrosis severity, whereas plasma phosphorous was not (Berchtold et al., 2016). Future work could investigate the effect of these hormones on feline renal cells in conjunction with increased extracellular phosphate.

## Conclusions

Feeding a phosphate-restricted renal diet to cats with CKD did not change urinary TGF-β1 concentration. Additionally, no association between increased extracellular phosphate and tubular cell apoptosis, tubular cell proliferation or expression of genes related to TGF-β1 signalling and ECM production was documented. These findings suggest the beneficial effects of dietary phosphate restriction on progression of feline CKD may not occur through modulation of renal TGF-β1 production, and do not support a direct pro-fibrotic effect of increased extracellular phosphate on feline renal cells.

## Conflict of interest

JSL was in receipt of a BBSRC CASE studentship co-funded by Elanco Ltd. at the time the study was carried out, but they played no role in the study design, in data collection, analysis and interpretation, or in the manuscript writing or submission for publication. JE is a member of the IRIS, which is sponsored by Elanco Ltd. None of the authors has any other financial or personal relationships that could inappropriately influence or bias the content of the paper.
