# Supplementary material for: Investigation of the transforming growth factor-beta 1 signalling pathway as a possible link between hyperphosphataemia and renal fibrosis in feline chronic kidney disease
Source: Vet J. 2021 Jan;267:105582. doi: 10.1016/j.tvjl.2020.105582 (PMC7814380; doi:10.1016/j.tvjl.2020.105582)
Supplement: Supplementary file 1 [file mmc1.docx]

**Table S1.** *P*-values for feline proximal tubular epithelial cell gene expression comparisons at different phosphate concentrations (0.95 mM vs. 2 mM vs. 3.5 mM) by one-way ANOVA. Post-hoc testing was not performed as ANOVA was non-significant in all cases.

| **Gene** | **24 h** | **72 h** | **168 h** |
| --- | --- | --- | --- |
| *TGF-β1* | 0.46 | 0.69 | 0.31 |
| *CTGF* | 0.93 | 0.84 | 0.82 |
| *COL1α1* | 0.56 | 0.83 | 0.15 |
| *FN1* | 0.71 | 0.23 | 0.83 |
| *CDH1* | 0.8 | 0.41 | 0.33 |
| *CDH2* | 0.10 | 0.36 | 0.12 |
| *ACTA2* | 0.75 | 0.70 | ND |
| *TG2* | 0.37 | 0.36 | ND |

*TGF-β1*, transforming growth factor-beta 1 gene; *CTGF*, connecting tissue growth factor gene; *COL1α1*, collagen type I alpha 1 gene; *FN1*, fibronectin gene; *CDH1*, E-cadherin gene; *CDH2*, N-cadherin gene; *ACTA2*, alpha-smooth muscle actin gene; *TG2,* transglutaminase 2 gene

**Table S2:** *P*-values for all chronic kidney disease feline cortical fibroblast gene expression comparisons by Student’s *t*-test

| Gene | 72 h |
| --- | --- |
| *TGF-β1* | 0.56 |
| *CTGF* | 0.62 |
| *COL1α1* | 0.57 |
| *FN1* | 0.69 |
| *ACTA2* | 0.83 |

TGF-β1, transforming growth factor-beta 1 gene; CTGF, connecting tissue growth factor gene; COL1α1, collagen type I alpha 1 gene; FN1, fibronectin gene; ACTA2, alpha-smooth muscle actin gene
